# Complete Genome Sequence and Annotation of a *Campylobacter jejuni* Strain, MTVDSCj20, Isolated from a Naturally Colonized Farm-Raised Chicken

**DOI:** 10.1128/genomeA.00852-14

**Published:** 2014-08-21

**Authors:** Michael E. Taveirne, Drew T. Dunham, William G. Miller, Craig T. Parker, Steven Huynh, Victor J. DiRita

**Affiliations:** a Department of Microbiology and Immunology, University of Michigan Medical School, Ann Arbor, Michigan, USA; b Produce Safety and Microbiology Research Unit, Agricultural Research Service, U.S. Department of Agriculture, Albany, California, USA

## Abstract

*Campylobacter jejuni* is a major cause of human food-borne illness, with contaminated poultry products serving as a main source of human infection. *C. jejuni* strain MTVDSCj20 was isolated from the cecal contents of a farm-raised chicken that was naturally colonized with *Campylobacter*. We present here the complete annotated genome sequence of MTVDSCj20.

## GENOME ANNOUNCEMENT

*Campylobacter jejuni* is a leading cause of bacterially derived human food-borne illness worldwide (1, 2). Human *Campylobacter*-derived gastroenteritis, campylobacteriosis, is predominantly caused by two species of *Campylobacter*, *C. jejuni* and *C. coli* (3, 4). *Campylobacter* infections are usually due to the consumption of contaminated poultry products, mainly chicken (5). The colonization of broiler chickens with campylobacters usually occurs after 2 weeks of age and can persist until slaughter, with high levels of campylobacters isolated from the ceca (up to 10^9^ CFU/g) (6).

In an effort to better understand the colonization mechanisms employed by campylobacters during a natural infection, Rainbow Ranger broiler chickens from a local farm in Dexter, MI, were tested for the presence of campylobacters. The cecal contents from 20 birds confirmed to be colonized with *Campylobacter* were harvested postslaughter, serially diluted in phosphate-buffered saline, and plated on *Campylobacter*-selective medium (Mueller-Hinton blood agar supplemented with vancomycin [40 µg/ml], cefoperazone [40 µg/ml], trimethoprim [10 µg/ml], and cycloheximide [100 µg/ml]). The colonies that arose on selective medium were confirmed to be *Campylobacter* by multiplex PCR using primers CJF/R (*C. jejuni hipO*), CCF/R (*C. coli glyA*), and 23SF/R (*C. jejuni* 23s rRNA) (7). A single *C. jejuni* isolate from bird 20 (strain MTVDSCj20) was restreaked and used for whole-genome sequence analysis.

Genome sequencing was performed using the shotgun reads obtained on an Illumina MiSeq desktop sequencer. A total of 3,437,992 reads with an average read length of 246 nucleotides (nt) were assembled *de novo* using the Roche Newbler assembler (version 2.6), resulting in 120 total contigs (>100 bp) and 70 large contigs (5 to 77 kb). A reference assembly against the *C. jejuni* NCTC 11168 genome was also performed within Newbler. The *de novo* large contigs and the contigs derived from the reference assembly were used to create a draft scaffold. The scaffold gaps were filled using the small-repeat *de novo* contigs and the Perl script Contig_extender3 (8). The final genome sequence had a coverage of 512×. Homopolymeric GC tracts were characterized using the high-depth MiSeq reads.

Protein-, rRNA- and tRNA-coding genes were identified as described previously (8). The genome was annotated based on those of the *C. jejuni* strains NCTC 11168 and 81-176 (accession no. AL111168.1 and CP000538.1, respectively). Additional annotation was performed using Artemis (9), the identification of Pfam domains (version 26.0 [10]), and BLASTp comparisons to proteins in the NCBI nonredundant database.

The complete annotated genome sequence is 1.65 Mbp and contains 1,618 open reading frames. The MTVDSCj20 genome contains an additional 22 fragmented coding sequences (CDSs), identified as pseudogenes. BLASTp analysis against the proteins predicted to be encoded by the *C. jejuni* NCTC 11168 and 81-176 genomes indicated that 1,462 (90%) of the MTVDSCj20 CDSs have orthologs in either the 11168 or 81-176 genomes. Most of the variable genes in MTVDSCj20 were contained within five regions: two regions encoding putative type I restriction/modification systems, the lipooligosaccharide (LOS) and capsular biosynthetic regions, and a genomic island linked to an arginyl-tRNA.

### Nucleotide sequence accession number.

The whole-genome sequence, assembly, and annotation of *C. jejuni* strain MTVDSCj20 have been deposited in GenBank under the accession no. CP008787.
